# Comparative Transcriptomic Analysis of Hu Sheep Pituitary Gland Prolificacy at the Follicular and Luteal Phases

**DOI:** 10.3390/genes13030440

**Published:** 2022-02-27

**Authors:** Zhen Wan, Hua Yang, Yu Cai, Jianyu Ma, Peiyong Cheng, Zhibo Wang, Feng Wang, Yanli Zhang

**Affiliations:** Jiangsu Livestock Embryo Engineering Laboratory, College of Animal Science and Technology, Nanjing Agricultural University, NO. 1 Weigang, Nanjing 210095, China; 2019105038@njau.edu.cn (Z.W.); 2018205011@njau.edu.cn (H.Y.); 2018105027@njau.edu.cn (Y.C.); 2020205016@njau.edu.cn (J.M.); 2020105038@stu.njau.edu.cn (P.C.); 2020205010@stu.njau.edu.cn (Z.W.); caeet@njau.edu.cn (F.W.)

**Keywords:** lncRNA, pituitary gland, LHB, fecundity, follicular phase, luteal phase

## Abstract

The pituitary gland directly regulates the reproduction of domestic animals. Research has increasingly focused on the potential regulatory mechanism of non-coding RNA in pituitary development. Little is known about the differential expression pattern of lncRNAs in Hu sheep, a famous sheep breed with high fecundity, and its role in the pituitary gland between the follicular phase and luteal phase. Herein, to identify the transcriptomic differences of the sheep pituitary gland during the estrus cycle, RNA sequencing (RNA-Seq) was performed. The results showed that 3529 lncRNAs and 16,651 mRNAs were identified in the pituitary gland. Among of them, 144 differentially expressed (DE) lncRNA transcripts and 557 DE mRNA transcripts were screened in the follicular and luteal phases. Moreover, GO and KEGG analyses demonstrated that 39 downregulated and 22 upregulated genes interacted with pituitary functions and reproduction. Lastly, the interaction of the candidate lncRNA XR_001039544.4 and its targeted gene *LHB* were validated in sheep pituitary cells in vitro. LncRNA XR_001039544.4 and LHB showed high expression levels in the luteal phase in Hu sheep. LncRNA XR_001039544.4 is mainly located in the cytoplasm, as determined by FISH analysis, indicating that XR_001039544.4 might act as competing endogenous RNAs for miRNAs to regulate LHB. LncRNA XR_001039544.4 knockdown significantly inhibited LH secretion and cell proliferation. LncRNA XR_001039544.4 may regulate the secretion of LH in the luteal-phase pituitary gland via affecting cell proliferation. Taken together, these findings provided genome-wide lncRNA- and mRNA-expression profiles for the sheep pituitary gland between the follicular and luteal phases, thereby contributing to the elucidation of the molecular mechanisms of pituitary function.

## 1. Introduction

Hu sheep, a famous sheep breed in China, are characterized by high fertility, year-round estrus and fast growth. Therefore, Hu sheep have become an ideal model with which to explore the mechanism of high fecundity in sheep breeding. To date, many studies have shown that sheep fecundity is affected by multiple genes, including mutations in *bone morphogenetic protein 15* (*BMP15*) [1], *growth differentiation factor 9* (*GDF9*) [2], *bone morphogenetic protein receptor type 1B* (*BMPR-1B*) [3] and *Booroola fecundity* (*FecB*) [4]. Among them, *FecB* is the first major gene associated with multiple birth traits in sheep, which can increase the ovulation rate and litter size [5,6]. The *FecB* gene has been found in many prolific Chinese sheep breeds, such as Hu sheep, Duolang sheep and Little Tail Han (STH). It has been confirmed that *FecB* gene mutation plays a vital role in increasing the ovulation rate and prolificacy in ewes, and the frequency of the *FecB* allele in Hu sheep is up to 53% [7]. In addition, reproductive traits in less-prolific sheep can be improved by crossbreeding with sheep carrying the *FecB* gene. Therefore, only Hu sheep with FecB BB genotypes were selected in this study.

Long non-coding RNAs (lncRNAs) are non-coding RNAs with a length greater than 200 nt that are transcribed by RNA polymerase II, which cannot encode proteins [8]. Recent studies have shown that lncRNAs are involved in many important physiological processes in mammals, such as testicular maturation [9], oocyte maturation [10], embryo implantation [11] and gonadotropin secretion [12], which have become a hot spot in genetics research [13]. With the rapid development of RNA-Seq technology in the field of animal genetics and breeding [14,15], many lncRNAs that affect reproduction process have been initially investigated [16,17,18]. For example, Danila Cuomo et al. [19] screened 636 lncRNAs that were mainly enriched with PI3K-Akt and TGF-β signaling pathways, which were closely related to the regulation of follicle development and steroid-hormone biosynthesis. Chunyan Li et al. [20] identified six lncRNAs in the sheep pineal gland between the follicular phase and luteal phase, which could participate in hormone activities to affect sheep reproductive performance via regulation of its target genes. Yang et al. [9] identified some lncRNAs, and their target genes were enriched with male-gonad-development signaling pathways. Furthermore, lncRNAs have been identified as key regulators in the hypothalamic–pituitary–gonadal axis that is associated with reproduction. The previous research on the pituitary mainly focused on pituitary tumors, such as lncRNA H19 [21]. However, the study of lncRNAs in the normal pituitary is still limited. More recently, many lncRNAs were identified in the pituitary gland and found to have potential roles in regulating pituitary function. In mice, MIR205HG can bind to Zbtb20 to regulate the transcriptional activity of PIT1, thereby regulating the secretion of growth hormone and prolactin in the anterior pituitary [22]. LncRNA TCONS_00066406 participates in the development of immature and mature sheep pituitary glands by regulating *Hydroxysteroid 17-β Dehydrogenase 12 (HSD17B12)* [23]. LncRNA MSTRG.259847.2 was confirmed to be associated with high and low prolificacy of Hu sheep [12]. These studies indicated the presence and role of lncRNA in the pituitary gland. However, studies on the function of sheep lncRNA in the follicular phase and luteal phase are limited.

Therefore, this study focused on analyzing transcriptomics in two stages of the pituitary gland in Hu sheep with *FecB BB* genotypes: the follicular phase (H-F) and luteal phase (H-L). DE lncRNAs were subjected to gene-function analysis and constructed into a lncRNA–mRNA interaction network. More importantly, we identified the interaction of the candidate lncRNA XR_001039544.4 with its target gene LHB in vitro and explored the potential effect of lncRNA XR_001039544.4 downregulation on pituitary function. This study aimed to explore the candidate regulators of sheep prolificacy at the transcriptional level, which is essential for better understanding the molecular mechanisms by which lncRNAs regulate sheep reproduction at different physiological stages, and for providing insight into other animals.

## 2. Materials and Methods

### 2.1. Ethics Statement

The animal study was approved by the Animal Care and Use Committee of Nanjing Agricultural University and was strictly conducted under the Animal Experiments guidelines (Approval ID: SYXK2011-0036).

### 2.2. Animals and Samples Collection

Six healthy female Hu sheep with *FecB* BB genotypes (*N* = 6, litter size = 3) were selected from the Taizhou Sheep Farm (Jiangsu, China). All non-pregnant ewes, approximately 2–3 years old with similar weights, were provided with free access to water and feed under natural lighting.

Before the experiment, all sheep were implanted with vaginal sponge (CIDR) for 11 days, then the vaginal sponge was removed and the removal time was set to 0 h. A total of 6 sheep were divided into follicular-phase (H-F) and luteal-phase (H-L) groups. Then, three sheep were slaughtered at 45 h (follicular phase). The other three sheep were slaughtered on the 9th day after CIDR removal (luteal phase). Pituitary samples were immediately collected and stored at −80 °C for further analysis after slaughtering.

### 2.3. RNA Extraction, Strand-Specific Library Construction and Sequencing

The total RNA of pituitary samples was extracted using the Trizol reagent (Invitrogen, 15596026, Carlsbad, CA, USA) according to the manufacturer’s protocol. RNA purity, quality and concentration assays, strand-specific library construction and sequencing were performed as previously described [23] and sequenced using Illumina Novaseq6000 by Gene Denovo Biotechnology Co (Guangzhou, China). All sequencing data were outsourced to GENE DENOVO (Guangzhou, China).

### 2.4. Data Analysis and Transcript Assembly

To obtain high-quality clean reads, filtering of the reads was performed as previously described. Then, the clean reads were mapped to reference genome Oar_ v1.0 by TopHat2 [24] (version 2.1.1, Johns Hopkins University, Baltimore, MD, USA). The reconstruction of transcripts was carried out with Stringtie software (version 1.3.4) and the HISAT2 program (version 2.1.0) [25,26], and the transcripts with ≥200 bp in length and ≥2 exons were filtered. All reconstructed transcripts were aligned with the reference genome to identify novel transcripts.

Two software programs, CNCI [27] (version 2) and CPC [28] (version 0.9-r2) (http://cpc.cbi.pku.edu.cn/ (accessed on 8 June 2021) were used to assess the protein-coding potential of novel transcripts by default parameters. According to the location of lncRNAs in the genome, lncRNAs were divided into five types: bidirectional lncRNAs, intergenic lncRNAs, intronic lncRNAs, antisense lncRNAs and sense-overlapping lncRNAs.

### 2.5. Analysis of Differential Expression of mRNAs and Long Noncoding RNAs

The DE transcript mRNAs and lncRNAs were analyzed by DESeq2 [29] software between two different groups. The false-discovery rate (FDR) < 0.05 and absolute fold change (FC) ≥ 1.5 were the criteria for identifying differentially expressed genes (DEGs).

### 2.6. Functional Enrichment Analysis of DEGs

To identify significantly enriched metabolic pathways or signal pathways in DEGs, the calculation of p-values with FDR correction was performed, taking FDR ≤ 0.05 as a threshold. The GO term or pathway with FDR ≤ 0.05 was defined as a significantly enriched pathway in DEGs.

### 2.7. LncRNA-mRNA Interaction Analysis

We predicted the targeted genes of lncRNAs according to antisense, cis-act and trans-act analysis. The complementary correlation of antisense lncRNA and mRNA was predicted by RNAplex (http://www.tbi.univie.ac.at/RNA/RNAplex.1.html (accessed on 8 June 2021). LncRNAs that cis-regulate with their neighboring genes within 10 kb on the same allele were identified as cis-lncRNAs. The trans-regulation lncRNAs were based on the correlation coefficient between lncRNAs and mRNAs, and values > 0.9 were considered trans-acting. Based on these three interaction relationships, pituitary-function-related genes and potential lncRNAs were filtered and constructed into a gene co-expression network.

### 2.8. Cell Transfection and qRT-PCR Verification

To validate the potential role of candidate DE lncRNAs on pituitary function, primary pituitary cells were isolated on the basis of previous studies [12]. The siRNA of lncRNA XR_001039544.4 was synthesized by the GenePharma company (Shanghai, China). The sequences of siRNA are listed in Table S1.

Pituitary cells were seeded into 6-well plates for siRNA transfection. When pituitary cells reached 60–70% confluence, the siRNA was mixed with the Lipofectamine 3000 reagent (Invitrogen, No. L3000008, Carlsbad, CA, USA) and added to 6-well plates seeded with pituitary cells, according to the manufacturer’s protocol. The cells were harvested 24 h after transfection for RNA extraction. qPCR was performed as previously described [23]. The primer sequences are presented in Table S1.

### 2.9. FISH Analysis

Using the lncRNA XR_001039544.4 FISH probe mix (Cy3 labeled) for RNA FISH analysis to assess lncRNA localization in pituitary cells with a FISH kit (GenePharma, F12201/50, Shanghai, China), nuclei were stained with DAPI (Beyotime, C1002, Shanghai, China). The 18S was used as a positive control for the cytoplasm. The specific experimental steps followed the manufacturer’s instructions. Images were captured using an LSM 710 laser scanning confocal microscope.

### 2.10. ELISA Assay

Cell supernatant was collected, and LH was determined using the ELISA assay (Amresco, DRE-S9371, Shanghai, China) after transfection, according to the manufacturer’s protocol. The sensitivity by this assay is 0.1 mIU/mL. The values of inter- and intra-variation coefficients for the kit were <10%.

### 2.11. Edu Assay

Cell proliferation was detected using kFluor555 Click-iT EdU Assay Kit (KGA337-500, KeyGEN BioTECH, Jiangsu, China), according to the manufacturer’s instructions.

### 2.12. Statistical Analysis

All experiments were repeated at least three times. Statistical analysis of data was performed using SPSS software (24.0 Edition, Chicago, IL, USA). The significance of differences between samples was assayed by *t*-test for two-group comparisons and by one-way ANOVA used for three or multiple groups. The data were expressed as mean + standard error (SEM). *p*-value < 0.05 (*) and *p*-value < 0.01 (**) indicate significant and extremely significant differences, respectively.

## 3. Results

### 3.1. Overview of Sequencing Data in Sheep Pituitary Gland

A total of 11.9 Gb of clean data were obtained in this study. About 73.4–84.5 million clean reads were obtained per sample of pituitary. The clean data of each sample reached 99% and the percentage of the Q30 base was higher than 90%. All data showed that the sequencing data were highly reliable. In addition, the GC content of the pituitary was 48.33% and 48.71% in the H-F and H-L groups, respectively. The average number of reads for the six samples reached 80,050,123, the ratios of mapped reads and unmapped reads were 95.46% and 4.54%, and more than 80% of the reads were uniquely mapped in both groups. The percentage of reads that mapped to the intron, exon and intergenic region were 61.76%, 23.59%, and 14.65%, respectively (Table S2).

### 3.2. Identification of LncRNAs and mRNAs in Hu Sheep Pituitary Gland

**Figure 1:**
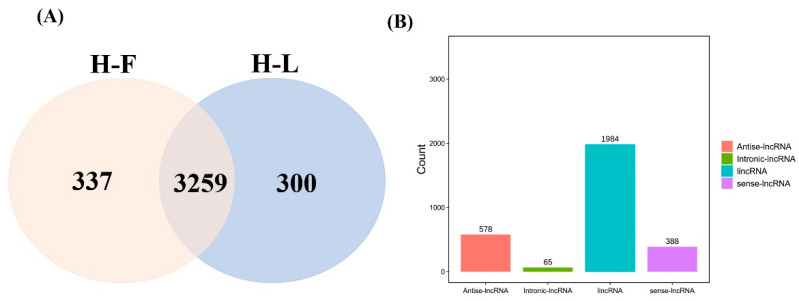
LncRNA identification and classification in the pituitary gland. (**A**) The lncRNAs were identified by two prediction software programs, CNCI and CPC. (**B**) The number statistics of different types of lncRNAs.

CPC/CNCI software was used to screen and identify lncRNAs after mapping to the reference genome (Figure 1A). We identified 3259 common lncRNAs, including 1984 lincRNAs (60.9%), 578 antisense lncRNAs (17.7%), 388 sense lncRNAs (11.9%) and 65 intronic lncRNAs (2.0%) (Figure 1B) and 16,651 mRNAs in pituitary gland.

**Figure 2:**
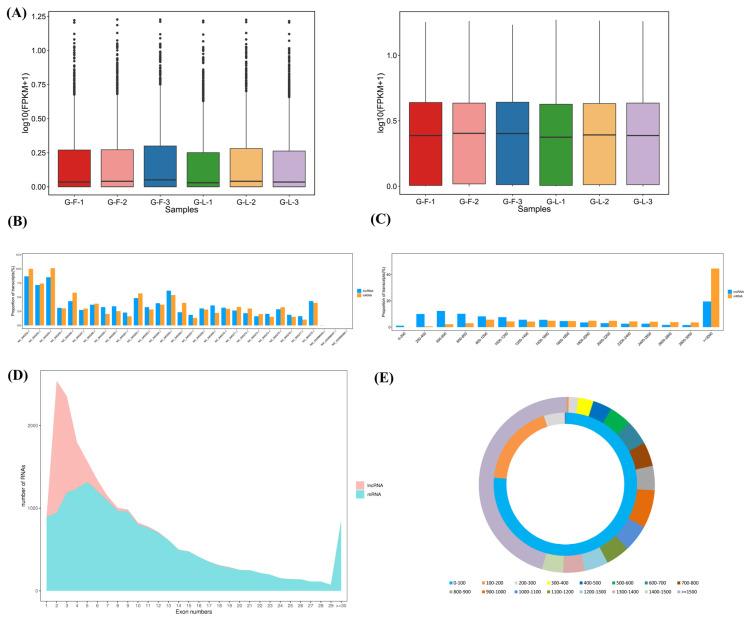
Comparison analysis of lncRNAs and mRNAs in sheep pituitary gland. (**A**) The boxplot shows the sample expression levels (log_10_ (FPKM)) of lncRNAs and mRNAs in the H-F and H-L groups. (**B**) The chromosome distribution of lncRNAs and mRNAs. (**C**) The length comparison of lncRNAs and mRNAs. (**D**) Exon content of lncRNAs and mRNAs. (**E**) Length of the open reading frame (ORF) of lncRNAs and mRNAs.

The expression level of lncRNAs was lower than mRNAs regardless of being in the H-F or H-L group (Figure 2A). As shown in Figure 2B, these lncRNAs and mRNAs were randomly assigned to the X-chromosome and 26 autosomes. The distribution trends of lncRNA and mRNA lengths were similar in the pituitary gland. The lncRNA and mRNA transcripts were mainly distributed over 3000 bp (Figure 2C). In addition, the majority of the lncRNAs had two exons while the mRNAs had five exons (Figure 2D). The open-reading-frame (ORF) length of the lncRNA transcripts was mainly concentrated within 100 bp, and the average ORF length of the lncRNA transcripts (about 76 bp on average) was shorter than that of the mRNA transcripts (about 953 bp on average, Figure 2E).

### 3.3. The Profiling and Verification of DE LncRNA and DEGs of Sheep Pituitary Gland

**Figure 3:**
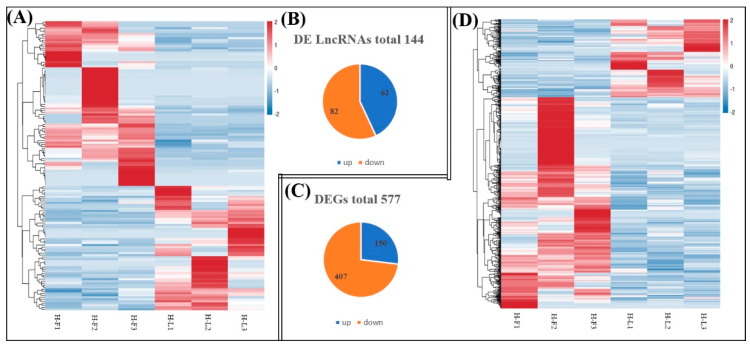
Comparisons of the number of DE lncRNAs and DE genes (DEGs) in H-F and H-L groups. (**A**) The hierarchical cluster of DE lncRNAs. (**B**) Total number of upregulated and downregulated DE lncRNAs in each comparison. (**C**) Total number of upregulated and downregulated DEGs in each comparison. (**D**) The hierarchical cluster of DEGs.

As shown in Figure 3, we identified 144 DE lncRNA transcripts (62 upregulated and 82 downregulated) and 557 DE mRNA transcripts (150 upregulated and 407 downregulated) between the H-F and H-L groups according to the criteria of FC > 1.5 and FDR < 0.05.

**Figure 4:**
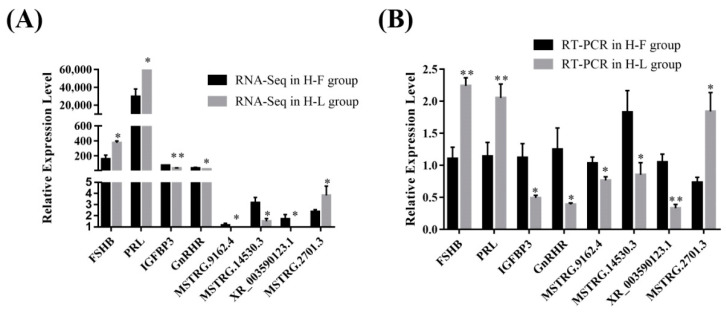
The verification of expression level of DE lncRNAs and DE mRNAs in different groups. (**A**) The relative expression level of four DE mRNAs and lncRNAs in different groups determined by RNA-Seq. (**B**) The relative expression level of four DE mRNAs and lncRNAs in different groups determined by RT-PCR (* *p* < 0.05; ** *p* < 0.01).

For further validation of the reliability of RNA-Seq data, four DE lncRNAs (MSTRG.5332.1, MSTRG.8834.3, MSTRG.14530.3, XR_003591934.1) and four DE mRNAs transcripts (IGFBP3, TP53INP1, FCF1, FSHB) were randomly selected and verified by using RT-qPCR (Figure 4). The results showed that the PCR results were consistent with the RNA-Seq results, which indicated that the data regarding lncRNA and protein-coding gene-transcript expression levels were reliable.

### 3.4. GO and KEGG Analysis of DEGs

**Figure 5:**
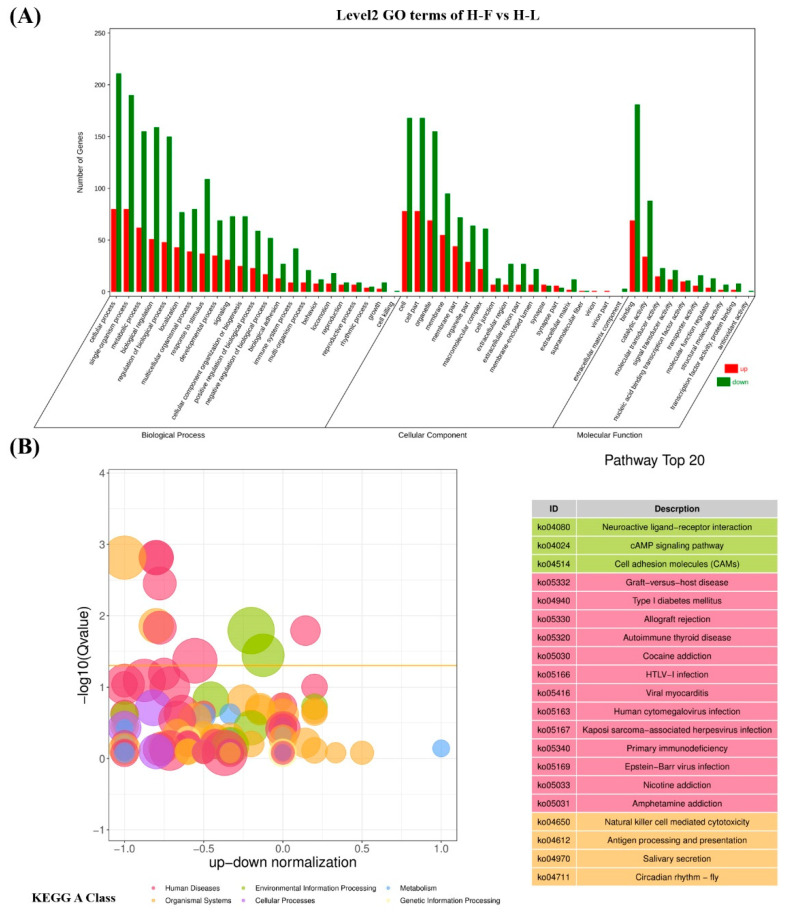
The top gene ontology (GO) and KEGG enrichment analysis. (**A**) The top 20 GO enrichment analysis of differential expressed genes (DEGs). (**B**) The top 20 KEGG enrichment analysis of DEGs.

All DEGs were tested in GO terms and subjected to KEGG enrichment analysis to clarify their underlying biological functions. In our study, 117 upregulated genes and 283 downregulated genes can be mapped to the GO database. The results showed that the most enriched GO term in the three parts were the cellular process, cell, and binding, respectively. As shown in Figure 5A, the top 20 most significantly enriched GO terms related to biological processes, including cellular process, single-organism process metabolic process, biological regulation, regulation of biological process, localization, multicellular-organismal process, response to stimulus, and so forth. In addition, GO terms related to pituitary function were enriched, including reproduction, reproductive process and growth (Table S3).

**Figure 6:**
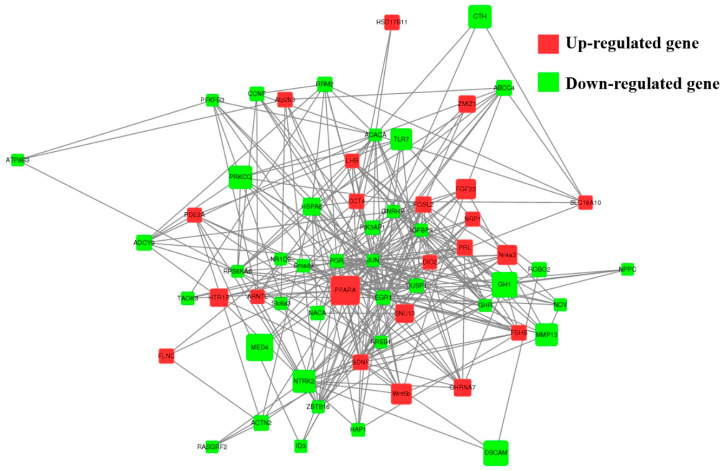
The network of 61 screened DE genes. The network of 61 screened DE genes enriched for Hu sheep pituitary functions and reproduction-related pathways were constructed, the red and green circles represent upregulated and downregulated DE genes, respectively. Node size represents the fold change of a node.

A total of 89 upregulated genes and 185 downregulated genes were annotated to 265 pathways according to KEGG enrichment analysis. As shown in Figure 5B, the highest enriched KEGG pathways in DEGs were mainly involved in the neuroactive ligand–receptor interaction, the cAMP signaling pathway and cell-adhesion molecules. Notably, some pathways were associated with pituitary function and reproduction, such as cAMP, PI3K-Akt, TGF-β, cGMP-PKG, MAPK, mTOR and serval hormone-related pathways (Figure S1). As shown in Figure 6, 39 downregulated and 22 upregulated genes that are associated with reproduction and pituitary functions were screened to construct the interaction network. The results showed that JUN, PGR, EGR1, DUSP1 and PPARA were the hub genes in the network.

### 3.5. LncRNA-Gene Interaction Network Construction

**Figure 7:**
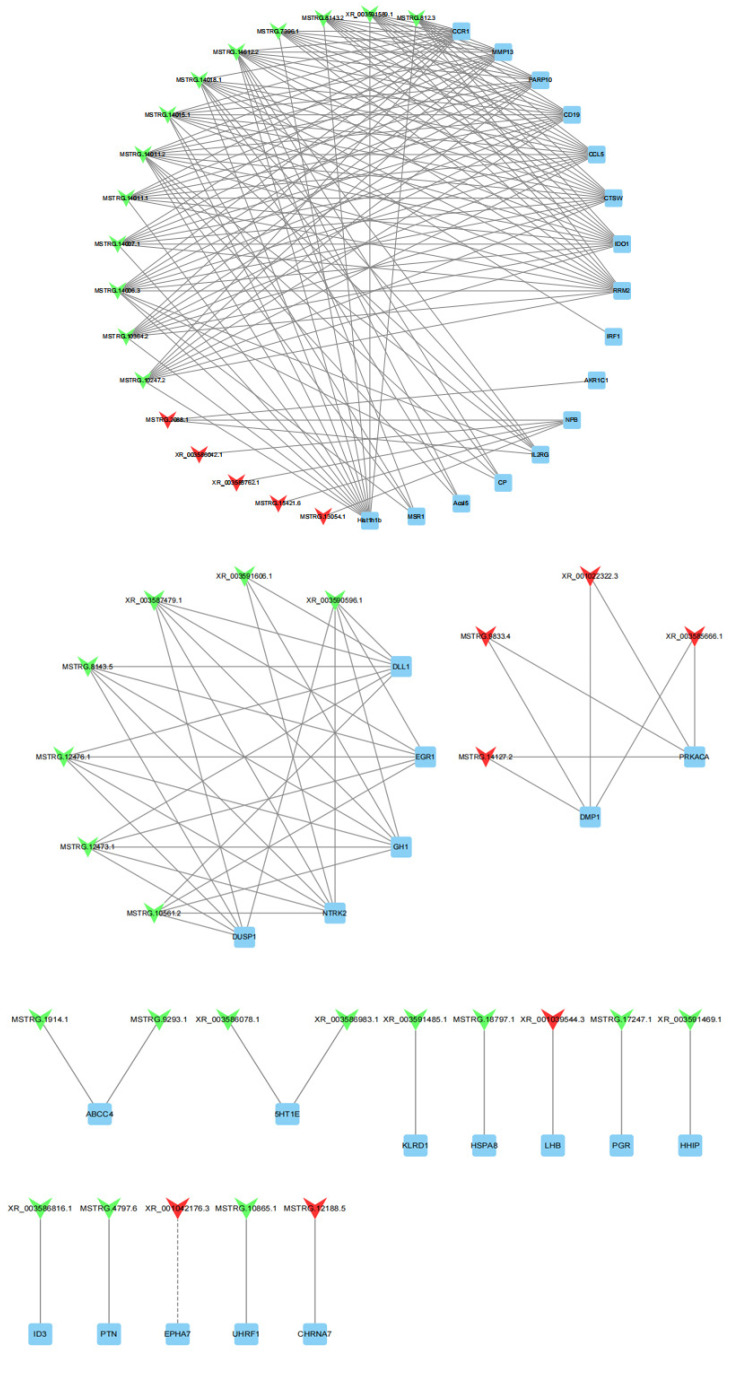
43 DE lncRNAs and their 36 predictably interactive cis- and trans-targeted genes comprised this interactive network. The red and green colors represent up and downregulation, quadrilaterals represent lncRNA, the box represents targeted genes, the straight line and dotted line represent the interaction relationship of trans- and cis-regulation, respectively.

To further explore the potential functions of lncRNAs in the sheep pituitary gland, we predicted 80 target genes of DE lncRNAs based on the cis and trans RNA–RNA-interaction principle. A total of 36 DE target genes enriched for sheep-reproduction-related pathways (Table S4), such as growth, ovarian steroidogenesis, reproduction, gonadotropin secretion, and cAMP, PI3K-Akt and MAPK signaling pathways were screened to construct lncRNA–gene co-expression network. One DE lncRNA and one cis-target, as well as 42 DE lncRNAs and 35 trans-targets were involved in this network (Figure 7). XR_001042176.3 was cis-acting with *EPHA7* by the sequence complementarity action. Meanwhile, XR_001039544.4 and MSTRG.17247.1 were trans-acting with *LHB* and *PGR*, respectively. MSTRG.10561.2 was trans-acting with *DLL1*, *EGR1*, *GH1*, *NTRK2* and *DUSP1*. The downregulated DE gene *IL2RG* was regulated by one upregulated lncRNA and four downregulated lncRNAs. Moreover, the DE genes *DMP1* and *PRKACA* were simultaneously regulated by four identical lncRNAs.

### 3.6. Verification and Characterization of XR_001039544.4 in Sheep Pituitary Cells

**Figure 8:**
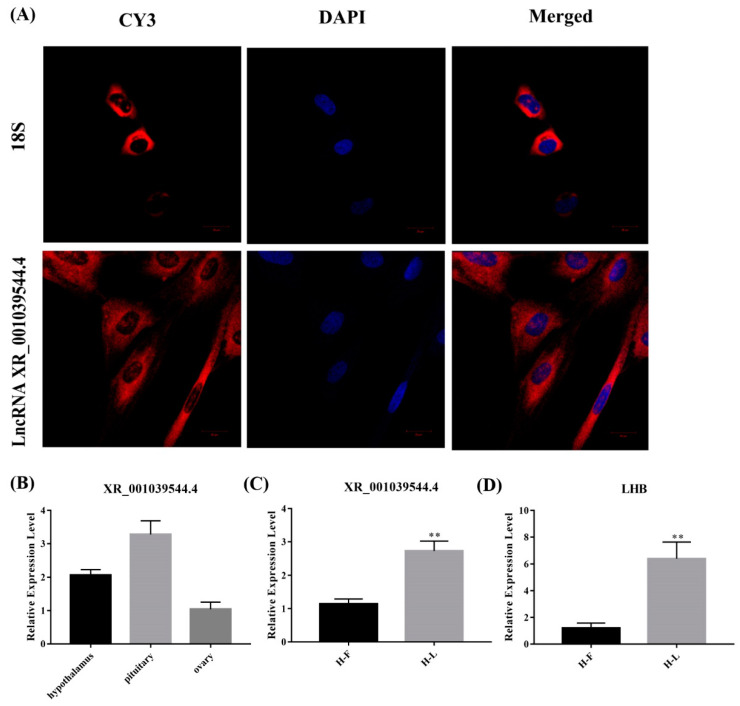
Identification of lncRNA XR_001039544.4. (**A**) lncRNA XR_001039544.4 localization in Hu sheep pituitary cells as detected by fluorescence in situ hybridization (FISH); scale bar, 20 μm. (**B**) Expression of lncRNA XR_001039544.4 in the Hu sheep hypothalamus, ovary and pituitary gland (HPO axis). (**C**,**D**) Expression of lncRNA XR_001039544.4 and its target gene *LHB* in different stages of Hu sheep pituitary gland. The data are shown as the mean ± SEM of at least three independent experiments. Statistical significance was analyzed by one-way ANOVA and Student *t*-test (** *p* < 0.01).

For validating the interaction between the lncRNAs and their target genes, and for exploring their potential function in pituitary cells, we screened lncRNA XR_001039544.4 for further experimentation, of which the target gene is *LHB*. First, the lncRNA XR_001039544.4 is an intergenic lncRNA located on chromosome 3. Then, we detected the localization of lncRNA XR_001039544.4 in pituitary cells, and a FISH experiment showed that lncRNA XR_001039544.4 was mainly located in the cytoplasm (Figure 8A). This suggests that lncRNA XR_001039544.4 may play a regulatory role by acting as a ceRNA regulatory mechanism. Further, we verified the expression of XR_001039544.4 and LHB in the pituitary at different periods, and qPCR results showed that lncRNA XR_001039544.4 and its target gene LHB were significantly increased the luteal phase (*p* < 0.01) (Figure 8C). Meanwhile, we detected the expression of lncRNA XR_001039544.4 in the HPO axis, and qPCR results showed that lncRNA XR_001039544.4 had the highest expression level in the pituitary gland (Figure 8B). Altogether, these data showed that lncRNA XR_001039544.4 could be a candidate lncRNA for exploring the molecular regulatory mechanisms of the luteal phase in the Hu sheep pituitary.

### 3.7. XR_001039544.4 Knockdown Downregulated the Secretion of LH in Pituitary Cells

**Figure 9:**
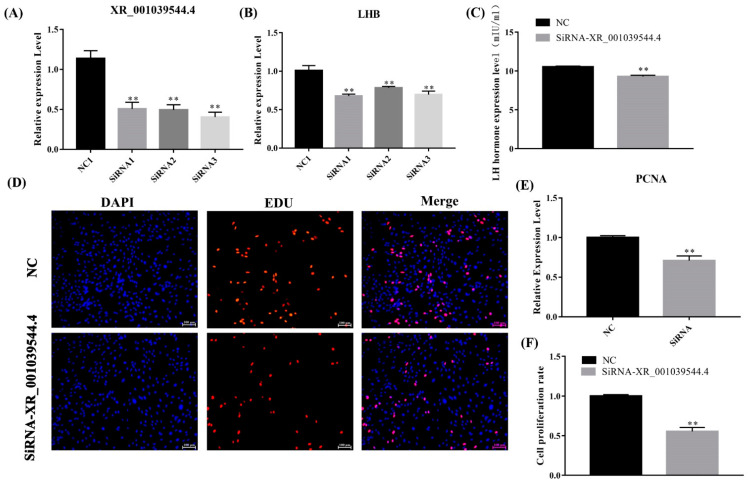
LncRNA XR_001039544.4 knockdown inhibits the secretion of gonadotropins in pituitary cells. (**A**,**B**) mRNA expression of lncRNA XR_001039544.4 and *LHB* in sheep pituitary cells treated with siRNAs were decreased compared to the negative control (NC), as determined by qPCR. (**C**) LH secretion was detected by ELISA. (**E**) mRNA expression of *PCNA* in lncRNA XR_001039544.4-knockdown pituitary cells. (**D**,**F**) Pituitary-cell proliferation was detected using EDU. Scale bars, 100 μm. The data are shown as the mean ± SEM of at least three independent experiments. Statistical significance was analyzed by one-way ANOVA and Student *t*-test (** *p* < 0.01).

To identify the function of lncRNA XR_001039544.4 in LH secretion, we transfected the sheep pituitary cells with lncRNA XR_001039544.4 siRNAs and detected the expression of XR_001039544.4 and *LHB*. The knockdown efficiency of siRNA1 was the highest among the three siRNAs. The expression level of its target gene, *LHB*, in the siRNA1-transfected group was significantly lower than in the control groups (Figure 9A,B). Furthermore, the ELISA results showed that the LH level significantly decreased (*p* < 0.05) in lncRNA XR_001039544.4-knockdown pituitary cells compared to the control group (Figure 9C). Overall, lncRNA XR_001039544.4 could regulate LH secretion by modulating LHB.

In addition, the *PCNA* mRNA expression level in the lncRNA XR_001039544.4-knockdown group was significantly lower than that in the control group (Figure 9E), and the Edu results showed that the cell-proliferation rate was significantly reduced (*p* < 0.01) in lncRNA XR_001039544.4-knockdown pituitary cells (Figure 9D,F). Taken together, these data indicated that XR_001039544.4 knockdown might significantly reduce LH secretion in pituitary cells by inhibiting cell proliferation.

## 4. Discussion

Reproductive traits are important factors affecting sheep production. In recent years, studies had investigated the mechanism of *FecB* affecting the reproductive performance of sheep [30,31,32,33]. *FecB* mutations can affect the response of oocytes to FSH and LH [34]. Increasing evidence also indicates that lncRNAs play important roles in sheep reproduction. However, the current research on lncRNA in the sheep pituitary gland is still relatively limited. FSH and LH secreted by the pituitary gland are key factors affecting follicular development and ovulation. Hu sheep are a Chinese indigenous breed with high fertility, and it is of great significance to comprehensively and deeply excavate the genes related to prolificacy. Therefore, we performed different lncRNA-expression profiles of the Hu sheep pituitary at different stages, including the follicular phase and the luteal phase, to explore the potential role of lncRNA in sheep prolificacy.

In this study, we investigated 3259 lncRNAs and 16,651 mRNAs in the sheep pituitary gland. We found that the chromosomal distribution trends of mRNAs and lncRNAs were similar. Notably, the distribution ratio of lncRNAs and mRNAs on chromosomes 1, 2 and 3 were greater than those on other chromosomes, which indicated that these three chromosomes may be closely related to pituitary function. Simultaneously, the length of lncRNAs and mRNAs showed the same trend. Moreover, the exon number, transcript and ORF length of mRNAs and lncRNAs in this study had a similar pattern to those in the immature and mature sheep pituitary gland [23]. In addition, we identified the expression levels of randomly selected lncRNAs, and the results were consistent with the RNA-Seq data. All the results suggested that the identified lncRNAs are reliable in the pituitary gland.

It is well known that the pituitary gland, one of the most important endocrine glands, can produce FSH, LH, GH and prolactin (PRL) hormones and so on. Overall, we screened 144 DE lncRNAs and 577 DE mRNAs during the follicular and luteal phases in the sheep pituitary gland. GO annotation and KEGG enrichment were performed on all transcripts. Among them, we found that hormone related genes *FSHB*, *LHB*, *PRL*, and *GH* were significantly differentially expressed. By the analysis of the interaction between lncRNAs and mRNAs, we finally found that there was one DE lncRNA that interacted with *LHB* and seven lncRNAs that interacted with *GH*. Based on the GO enrichment analysis, 61 DEGs were specifically enriched in reproduction, growth and pituitary function. *Smad9*, *RBP4*, *ZBTB16*, *CCNF*, *ATP8B3*, *CCT4*, *ROBO2*, *PGR* and *NPPC* were enriched in reproduction, which suggests that these genes might be associated with pituitary function in Hu sheep.

Moreover, the KEGG enrichment analysis showed that 23 signaling pathways were related to reproduction, such as the cAMP, GnRH, TNF, TGF-b and MAPK signaling pathway. Among them, the number of genes enriched in the cAMP signaling pathway was the largest. In the pituitary gland, the cAMP pathway plays an essential role in regulating cell proliferation and differentiation [35], as well as hormone secretion and production [36]. The cAMP pathway can also interact with the PI3K/AKT/mTOR, MAPK/ERK and WNT pathways to regulate the normal physiology of the pituitary gland [37]. Our study showed that these DE genes, such as *BDNF*, *PPARA*, *NTRK2*, *ADCY9*, and *WNT5B*, were involved in multiple signaling pathways at the same time. Studies have found that *BDNF* can participate in the regulation of the hypothalamic–pituitary–adrenal axis in mice. Knockout of *BDNF* in the mouse brain increases the release of adrenocorticotrophic hormone [38]. Meanwhile, *BDNF* and *NTRK2* induces neuronal hyperexcitability in TG neurons and pain hypersensitivity in rats by activating p38 MAPK and inhibiting the PI3K pathway [39]. It also means that these genes might be considered as candidate genes for further study on pituitary function. Interestingly, *EGR1*, *JUN*, *PGR*, and *PPARA* were at the core of the interaction networks. However, whether there are any further links between these key genes, and how they cooperate with each other remain largely unknown and need further study.

LncRNAs are a heterogeneous type of ncRNAs that have been shown to display crucial regulatory functions in some biological processes [40]. Therefore, we constructed the lncRNA-target-gene-interaction networks. In our study, XR_001042176.3 can cis-regulate *EPHA7*, which was upregulated at luteal phase. Previous research demonstrated the involvement of EPHA7-EFNA5 signaling in the regulation of the LH and E2 negative-feedback pathways in the hypothalamus [41], highlighting the functional role of *EPHA7* in female reproduction [42]. We also found that lncRNA MSTRG.8143.5, which was upregulated at the follicular phase, simultaneously targeted *DLL1*, *EGR1*, *GH1*, *DUSP1* and *NTRK2* in the sheep pituitary gland. The *early-growth-response* (*EGR1*) gene resides within the GnRH transcriptional network and belongs to the primary-response-gene family [43]. The detectable changes in EGR1 transcription occur as early as possible within 1 h of GnRH stimulation [44]. As the EGR1 protein accumulates, it regulates the transcription of secondary-like MAPK phosphatase 2 [45] and tertiary-like *LHB* response genes [46]. In addition, the JNK and ERK1/2 signaling pathway were involved in the GnRH induction of Egr1PT/mRNA in female-rat pituitary cells. Therefore, *EGFR1* and MSTRG.8143.5 may be important for pituitary function at the follicular phase [47].

In pituitary cells, LH is a heterodimeric glycoprotein that shares a common α subunit (α-GSU) with other glycoprotein hormones. However, the β-subunits are receptors and hence are hormone-specific [48]. LH and FSH act on gonadal cells to regulate gonadal growth, differentiation, steroid production and gametogenesis. Studies have found that LHB-knockout female mice are hypogonadal and exhibit decreased levels of serum estradiol and progesterone [49]. In our study, according to the networks, lncRNA XR_001039544.4 was trans-acting on its target gene *LHB*, which could be considered as a candidate gene to investigate its function in the pituitary. LncRNA XR_001039544.4 had the highest expression in the pituitary gland of the HPO axis. After knockdown of lncRNA XR_001039544.4, the expression of *LHB* was significantly decreased, indicating that lncRNA XR_001039544.4 might play a regulatory role through *LHB*. As a key factor in the regulation of gene expression, lncRNAs can produce different modes of action according to the subcellular localization pattern [50,51]. LncRNAs can act as cis or trans in the nucleus to silence or activate specific genes [52]. In contrast, lncRNAs appear in the cytoplasm and can participate in a variety of molecular mechanistic processes, including mRNA stability and translation regulation, and are mediated by protein modifications, miRNA precursors or competing endogenous RNAs [53]. The FISH experiment results showed that lncRNA XR_001039544.4 was mainly located in the cytoplasm, which means that it may act as competing endogenous RNAs (ceRNAs) for miRNAs to regulate *LHB* expression. By downregulating the expression of the lncRNA XR_001039544.4, the *PCNA* gene was found to be significantly reduced, indicating that lncRNA XR_001039544.4 could maintain proliferation in pituitary cells. Taken together, the DE lncRNAs identified in this study might cooperate with their target genes and DEGs to regulate pituitary functions.

## 5. Conclusions

In summary, the transcriptomic pituitary-gland study revealed the differential regulation of lncRNAs and mRNAs related to the follicular phase and luteal phase in Hu sheep with high prolificacy. We screened a set of lncRNAs and genes associated with reproduction and pituitary functions. According to the GO and KEGG databases, the DEGs were annotated with multiple physiological processes related to the pituitary gland such as hormone synthesis and regulation, growth, and the reproductive process. Additionally, we predicted the potential function of DE lncRNAs and constructed the lncRNA-gene-interaction network in this study to provide a valuable resource of candidate lncRNAs in the pituitary gland. Furthermore, these differential mRNA- and lncRNA-expression profiles provide valuable resources for determining the molecular mechanism of sheep prolificacy.

## Data Availability

All data in this study are available, if requested.

## Supplementary Materials

The following supporting information can be downloaded at: https://www.mdpi.com/article/10.3390/genes13030440/s1, Figure S1: Functional Enrichment Analysis for pituitary function and reproduction of DE mRNAs. Table S1: The list of primer sequences for lncRNAs and mRNAs. Table S2: Statistical results of reads in each pituitary sample. Table S3 The GO enrichment analysis of DE mRNAs. Table S4: The list of LncRNA–gene interaction (cis and trans RNA–RNA interaction).
