# Supplementary material for: Comparative Transcriptomic Analysis of Hu Sheep Pituitary Gland Prolificacy at the Follicular and Luteal Phases
Source: Genes (Basel). 2022 Feb 27;13(3):440. doi: 10.3390/genes13030440 (PMC8949571; doi:10.3390/genes13030440)
Supplement: Supplementary file 1 [file genes-13-00440-s001.zip › Tables/Table S1.pdf]

Table S1 The list of primer sequences for lncRNAs and mRNAs.

Gene symbol	Primer Sequence	Product size (bp)
ACTB	F: CGCAAGTACTCCGTGTGGAT<br>R: TAACGCAGCTAACAGTCCGC	146
MSTRG.9162.4	F: CTTAGTTCACTCGCTCTGTCCTGTC<br>R: TGGTTGGATGGCATCACTGACTTG	103
MSTRG.14530.3	F: TCGATGTTCAGGACAGCACC<br>R: CACGTGGAGACAGAAGCACT	209
XR_003590123.1	F: GTTGGACACGGATTCTCACTGACC<br>R: GACAGCAGAGGCGGTATTGACAG	143
MSTRG.2701.3	F: CTGCTCTGCCACCTTATTCTCCTTC<br>R: GCTGTGACCTGCCTTCTTCTCTG	103
PRL	F: AACACACTTCACCAGGACAGC<br>R: ATTGGGACAGACAGGGGTGG	120
IGFBP3	F: AAGGTCATGCCAAGGACAGC<br>R: AGTGTGTCCTCCATTTCCCG	133
GnRHR	F: TGGACGCCCTACTATGTCCT<br>R: GCTTTGCGTTTCTCATTCCC	224
XR_001039544.4	F: GCAGGACATGATGACTCTCCAAGTG<br>R: GCAGTACAGGTCGGACAACATAGC	150
FSH	F: GCTATTGCTACACCCGGGAC<br>R: AGTGGCTACTGGGTACGTGT	152
LH	F: GCGTGGACCCAATGGTCTCTTTC<br>R: ACAGGCCAAGGGTTGGGTTCTG	107
BAX	F: TTGGCTGAGTCGCTGAAGAGC<br>R: AACTCCCATGGCCCCCAAAT	228
BCL2	F: CCTTTCGTTTGCTCGTGCTC<br>R: ACCTCCTCCGTGATGTGGTAT	273
PCNA	F: CCTTGGTGCAGCTAACCCTT<br>R: GCCAAGGTGTCCGCATTATC	163
**Gene symbol**	**TCONS_00066406 siRNA sequence (5'-3')**	**Target sites**
**siRNA-NC**	sense: UUCUCCGAACGUGUCACGUTT<br>antisense: ACGUGACACGUUCGGAGAATT	Negative control
**SiRNA1-*XR_001039544.4***	sense: GGCAGGACAUGUGUCUUAUTT<br>antisense: AUAAGACACAUGUCCUGCCTT	379
**SiRNA2-*XR_001039544.4***	sense: GGGCACAUGUCUUAAUCAUTT<br>antisense: AUGAUUAAGACAUGUGCCCTT	535
**SiRNA3-*XR_001039544.4***	sense: CUGCCAAGCACAUGGUAAATT<br>antisense: UUUACCAUGUGCUUGGCAGTT	672
