# Supplementary material for: Comparative Transcriptomic Analysis of Hu Sheep Pituitary Gland Prolificacy at the Follicular and Luteal Phases
Source: Genes (Basel). 2022 Feb 27;13(3):440. doi: 10.3390/genes13030440 (PMC8949571; doi:10.3390/genes13030440)
Supplement: Supplementary file 1 [file genes-13-00440-s001.zip › Tables/Table S2.pdf]

Table S2. Statistical results of reads in each pituitary sample

Sample	Total Reads	Unmapped Reads	Unique Mapped Reads	Multiple Mapped reads	Mapping Ratio
**H-F1**	77925260	3918589 (5.03%)	67206634 (86.24%)	6800037 (8.73%)	74006671 (94.97%)
**H-F2**	84181856	3935036 (4.67%)	71870823 (85.38%)	8375997 (9.95%)	80246820 (95.33%)
**H-F3**	76516172	4022747 (5.26%)	66671541 (87.13%)	5821884 (7.61%)	72493425 (94.74%)
**H-L1**	77997756	3813754 (4.89%)	66113641 (84.76%)	8070361 (10.35%)	74184002 (95.11%)
**H-L2**	82377682	3421812 (4.15%)	70305891 (85.35%)	8649979 (10.50%)	78955870 (95.85%)
**H-L3**	79108326	2578152 (3.26%)	68454932 (86.53%)	8075242 (10.21%)	76530174 (96.74%)

1) H-F1, H-F2, H-F3 and H-L1, H-L2, H-L3 represent the follicular phase(H-F) and luteal phase(H-L) sheep pituitary sample with *FecB BB* genotype, respectively.
2) Total Reads: The number of clean reads.
3) Unmapped Reads: The number of sequences not matched to the genome.
4) Unique Mapped Reads: The number of sequences that had unique positions mapped to the reference sequence.
5) Multiple Mapped Reads: The number of sequences that had multiple positions mapped to the reference sequence.
6) Mapped Ratio: The number of sequences mapped to the reference sequence.
