# Supplementary material for: Comparative Transcriptomic Analysis of Hu Sheep Pituitary Gland Prolificacy at the Follicular and Luteal Phases
Source: Genes (Basel). 2022 Feb 27;13(3):440. doi: 10.3390/genes13030440 (PMC8949571; doi:10.3390/genes13030440)
Supplement: Supplementary file 1 [file genes-13-00440-s001.zip › Tables/Table S3.pdf]

Table S3. The GO enrichment analysis of DE mRNAs.

Function	GO terms	Gene number	UP-regulated	DOWN-regulated	p value
Hormone synthesis and regulation	response to hormone	15	5	10	0.066541
	cellular response to hormone stimulus	8	4	4	0.113196
	cellular response to steroid hormone stimulus	4	1	3	0.180815
	response to steroid hormone	7	2	5	0.196335
	regulation of hormone secretion	2	1	1	0.565468
	regulation of hormone levels	2	1	1	0.864904
	negative regulation of hormone secretion	1	1	0	0.054602
	steroid metabolic process	3	2	1	0.827277
	negative regulation of gonadotropin secretion	1	1	0	0.027683
	regulation of secretion by cell	8	5	3	0.074595
Growth	growth	12	3	9	0.840975
	regulation of developmental growth	4	1	3	0.17531
	tissue development	35	12	23	0.00999
	cell proliferation	31	5	26	0.262762
	positive regulation of cell growth	2	1	1	0.221667
	animal organ development	53	15	38	0.015193
	fibroblast growth factor receptor signaling pathway	1	1	0	0.265771295
	negative regulation of developmental growth	1	1	0	0.265771
	multicellular organism growth	2	0	2	0.293933
	positive regulation of growth	2	1	1	0.385166
	developmental growth	5	1	4	0.434364
	response to growth factor	7	4	3	0.493942
	regulation of growth	9	2	7	0.543751
	negative regulation of growth	1	1	0	0.615485
	cell growth	5	1	4	0.740214
Reproduction	multi-organism reproductive process	10	5	5	0.623619
	multicellular organismal reproductive process	7	3	4	0.732346
	single organism reproductive process	13	5	8	0.748638
	reproductive process	16	7	9	0.780522
	reproductive structure development	1	0	1	0.975201
	negative regulation of reproductive process	1	0	1	0.223334
	reproductive system development	1	0	1	0.975201
	regulation of reproductive process	1	0	1	0.3969
	sexual reproduction	9	4	5	0.633391
	multicellular organism reproduction	8	3	5	0.63742
	reproduction	16	7	9	0.791815
	developmental process involved in reproduction	6	1	5	0.876677
	cellular process involved in reproduction in multicellular organism	3	0	3	0.434336

	Wnt signaling pathway	1	1	0	0.994346
	development of primary sexual characteristics	1	0	1	0.918522
	gonad development	1	0	1	0.91131
	Notch signaling pathway	3	1	2	0.14702
