# Supplementary figures and images for: Comparative Transcriptomic Analysis of Hu Sheep Pituitary Gland Prolificacy at the Follicular and Luteal Phases
Source: Genes (Basel). 2022 Feb 27;13(3):440. doi: 10.3390/genes13030440 (PMC8949571; doi:10.3390/genes13030440)

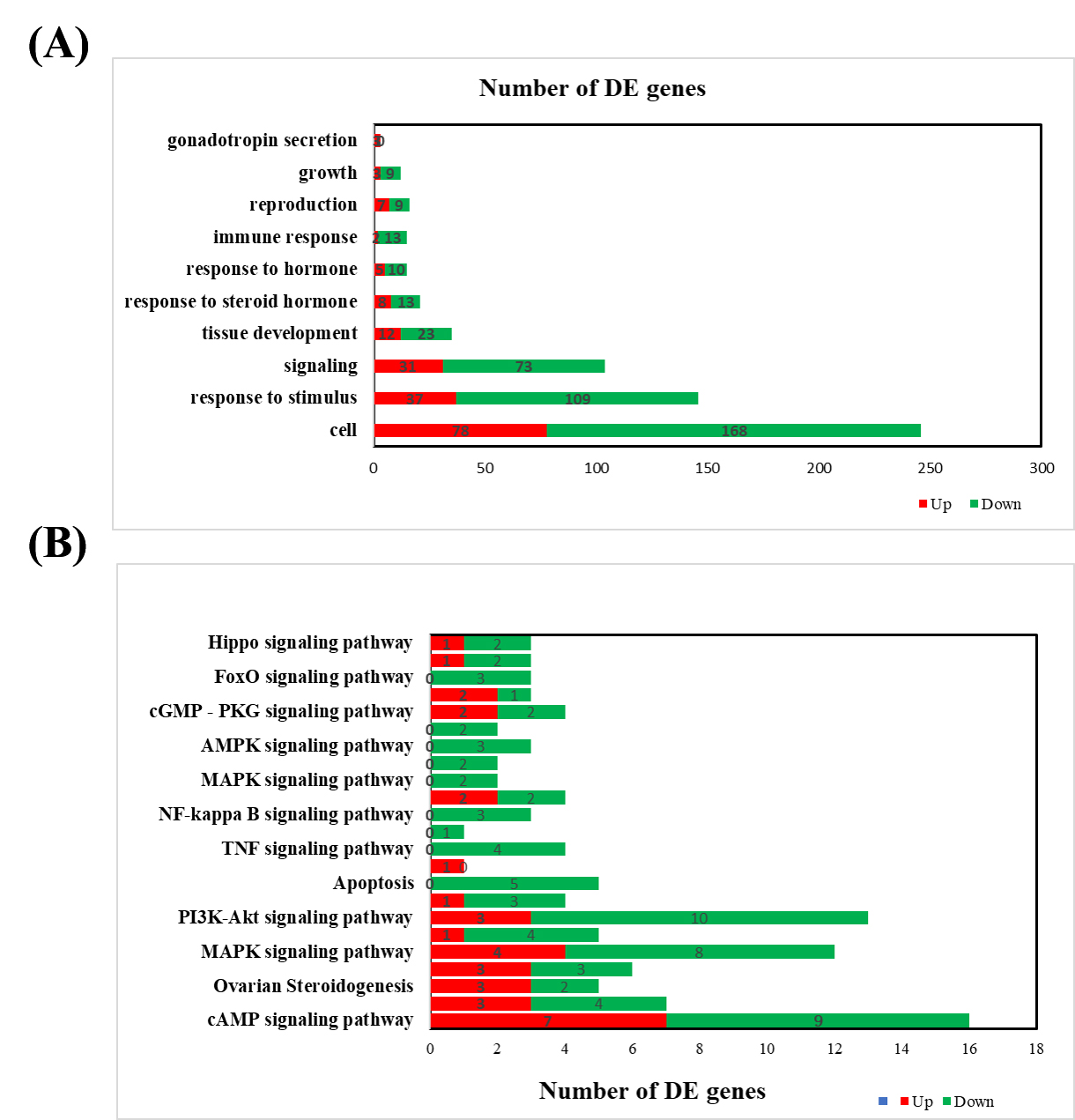

Supplement: Supplementary file 1 [file genes-13-00440-s001.zip › Figure S1.jpg]
